# 9G Test^TM^ Cancer/Lung: A Desirable Companion to LDCT for Lung Cancer Screening

**DOI:** 10.3390/cancers12113192

**Published:** 2020-10-30

**Authors:** Wonho Choe, Jeong Don Chae, Byoung-Hoon Lee, Sang-Hoon Kim, So Young Park, Satish Balasaheb Nimse, Junghoon Kim, Shrikant Dashrath Warkad, Keum-Soo Song, Ae-Chin Oh, Young Jun Hong, Taisun Kim

**Affiliations:** 1 Nowon Eulji Medical Center, Department of Laboratory Medicine, Eulji University, Seoul 01830, Korea; choewh@eulji.ac.kr (W.C.); jdchae@eulji.ac.kr (J.D.C.); 2 Nowon Eulji Medical Center, Department of Pulmonology and Allergy, Eulji University, Seoul 01830, Korea; hoonakr@eulji.ac.kr (B.-H.L.); ksh1134@eulji.ac.kr (S.-H.K.); soyoung.park@eulji.ac.kr (S.Y.P.); 3 Institute of Applied Chemistry and Department of Chemistry, Hallym University, Chuncheon 24252, Korea; satish_nimse@hallym.ac.kr (S.B.N.); hunsjung@hallym.ac.kr (J.K.); 4 Biometrix Technology, Inc. 2-2 Bio Venture Plaza 56, Chuncheon 24232, Korea; shrikant.warkad@hallym.ac.kr (S.D.W.); hanlimsk@empal.com (K.-S.S.); 5 Departments of Laboratory Medicine, Korea Cancer Center Hospital, Seoul 01812, Korea; 2965@kirams.re.kr

**Keywords:** lung cancer, low-dose computed tomography, LDCT, biomarkers, CYFRA 21-1, cancer screening, biopsy, stage I cancers, neoplasm, non-invasive cancer diagnostics, auto-antibody

## Abstract

**Simple Summary:**

Lung cancer is the most common cause of cancer-related deaths globally. Patients diagnosed at early-stage (0–I) have a higher survival rate than the metastasized stages (III–IV). Thus, there is great potential to reduce mortality by diagnosing lung cancer at stage 0~I through community screening. LDCT is a promising method, but it has a high false-positive rate. Therefore, a biomarker test that can be used in combination with LDCT for lung cancer screening to reduce false-positive rates is highly awaited. The present study evaluated the applicability of 9G test^TM^ Cancer/Lung test to detect stage 0~IV lung cancer. 9G test^TM^ Cancer/Lung test detects stage I, stage II, stage III, and stage IV cancers with the sensitivities of 77.5%, 78.1%, 67.4%, and 33.3%, respectively, at the specificity of 97.3%. These results indicate that the 9G test^TM^ Cancer/Lung can be used in conjunction with LDCT to screen lung cancer.

**Abstract:**

A complimentary biomarker test that can be used in combination with LDCT for lung cancer screening is highly desirable to improve the diagnostic capacity of LDCT and reduce the false-positive rates. Most importantly, the stage I lung cancer detection rate can be dramatically increased by the simultaneous use of a biomarker test with LDCT. The present study was conducted to evaluate 9G test^TM^ Cancer/Lung’s sensitivity and specificity in detecting Stage 0~IV lung cancer. The obtained results indicate that the 9G test^TM^ Cancer/Lung can detect lung cancer with overall sensitivity and specificity of 75.0% (69.1~80.3) and 97.3% (95.0~98.8), respectively. The detection of stage I, stage II, stage III, and stage IV cancers with sensitivities of 77.5%, 78.1%, 67.4%, and 33.3%, respectively, at the specificity of 97.3% have never been reported before. The receiver operating characteristic curve analysis allowed us to determine the population-weighted AUC of 0.93 (95% CI, 0.91–0.95). These results indicate that the 9G test^TM^ Cancer/Lung can be used in conjunction with LDCT to screen lung cancer. Furthermore, obtained results indicate that the use of 9G test^TM^ Cancer/Lung with LDCT for lung cancer screening can increase stage I cancer detection, which is crucial to improve the currently low 5-year survival rates.

## 1. Introduction

Lung cancer is the most common cause of cancer-related deaths globally, accounting for about 18.4% of total cancer deaths [1]. In 2018 alone, around 2 million individuals were identified with lung cancer, and nearly 1.7 million deaths were reported due to lung cancer on a global scale [2]. Most lung cancer cases are diagnosed at advanced stages because of the asymptomatic nature of lung neoplasms. Unfortunately, in cases of advanced lung cancer, cure with current therapies is unlikely. The 5-year survival rate in lung cancer is meager. However, patients diagnosed at an early stage have a higher survival rate than the latter, localized, and metastasized stages (54.8% vs. 27.4% vs. 4.2%, respectively) [3,4]. The screening and diagnosis of lung cancer at an early stage saves lives [5]. Thus, there is great potential to reduce mortality by diagnosing lung cancer at stage 0~I through community screening. 

Though community screening is vital to identify lung cancer patients at stage 0~I, overdiagnosis in terms of false-positive results is a troubling aspect of cancer screening [6,7,8]. Conventionally, chest X-ray (CXR) and sputum cytology are used for the screening for lung neoplasms. However, conventional screening methods are ineffective in detecting early-stage lung cancer [9,10]. Low-dose computed tomography (LDCT) has emerged as a promising mass screening method for the early diagnosis of lung neoplasms [11]. The advantage of the LDCT-based protocol is its simplicity and its high sensitivity [12,13]. The Early Lung Cancer Action Project (ELCAP) showed that the LDCT scans were six times higher in accuracy and sensitivity than CXR [14]. The DANTE study also confirmed that the LDCT has high screening efficacy in detecting stage I lung cancers [15].

The National Lung Screening Trial (NLST), reported in 2011, compared LDCT with CXR, established a 20% reduction in lung cancer mortality, and a 7% reduction in all-cause mortality in favor of LDCT [16]. However, after three screening rounds, 25% of subjects were classified as positive, with 96% of them confirmed as false-positive upon biopsy [17]. In this regard, the detection of biomarkers circulating in plasma or serum offers excellent potential for developing non-invasive cancer diagnostics to detect stage 0~IV lung cancers [18,19]. Therefore, a complementary biomarker test that can be used in combination with LDCT for lung cancer screening to improve the diagnostic capacities and reduce false-positive rates is highly awaited. Most importantly, the stage I lung cancer detection rate can be dramatically increased by the simultaneous use of the biomarker test with LDCT.

There are several reports on using various biomarkers, including metabolites [20,21], volatile compounds [22], cDNA [23], miRNA [24], circulating tumor cells [25], antigens, and autoantibodies [26] to detect lung cancer. It is expected that these methods should reduce the false-positive rate when used in combination with LDCT. However, according to the reports, these methods suffer from low sensitivity (10~60%) and specificity (52~86%) for detecting lung cancer [27,28,29]. Therefore, a high sensitivity and specificity method is highly awaited to complement the LDCT in community-based lung cancer screening.

According to recent reports, cardiac troponin T (cTnT) levels are higher in treatment-naïve cancer patients than healthy people [30,31]. Increased cTnT levels are related to the cancer-associated arterial micro-thrombosis promoted by neutrophil extracellular traposis [32] and due to the elevated levels of cancer-specific autoantibodies [33,34]. The increased levels of autoantibodies are frequently associated with the micro-thrombosis [35]. The N-terminal pro-brain natriuretic peptide (NT-proBNP) is also considered a valuable marker for the prognosis of the oncologic disease [36].

We have recently reported on the quantification of CYFRA 21-1 and CYFRA 21-1-Anti-CYFRA 21-1 autoantibody immune complex (CIC) to detect early-stage lung cancer [37]. Based on our findings, we have developed a 9G test^TM^ Cancer/Lung, a test that allows the diagnosis of stage 0~IV lung cancers. 9G test^TM^ Cancer/Lung, which has high applicability in the health care programs for lung cancer screening as a supplementary to the LDCT, detects four biomarkers in the plasma samples.

To date, several studies were focused on the efforts to reduce the false-positive rate of LDCT by using a companion biomarker-based test. However, due to the reported methods’ low sensitivity and specificity, they are not suitable as companion tests to LDCT. Therefore, the present study was conducted to evaluate 9G test^TM^ Cancer/Lung tests sensitivity and specificity to detect Stage 0~IV lung cancer. The obtained results indicate that the 9G test^TM^ Cancer/Lung can detect lung cancer with overall sensitivity and specificity of 79.8% (74.2–84.7%) and 96.0% (92.6–98.2%), respectively. These results indicate that the 9G test^TM^ Cancer/Lung has a high potential for a community screening of lung cancer in conjunction with LDCT. Further, obtained results indicate that the use of 9G test^TM^ Cancer/Lung with LDCT for lung cancer screening can increase stage I cancer detection, which is crucial to improve the currently meager 5-year survival rates.

## 2. Results

The cohort was composed of clinical samples (*n* = 603) from healthy individuals (*n* = 359) and lung cancer patients (*n* = 244) collected and tested at various locations in South Korea (Table 1). All samples were collected by following the institutional guidelines. The respective IRB numbers are presented in Table 1.

All clinical samples (*n* = 603) were previously screened with the LDCT. Overall, 266/603 samples and 337/603 samples were found to be LDCT positive and LDCT negative, respectively. A biopsy was performed on the LDCT positive samples to confirm lung cancer. In total, 22/266 LDCT positive samples were found to be lung cancer negative in the biopsy. Of the LDCT positive samples, 244/266 were confirmed to have lung cancers and lung cancer stages, including stage I (*n* = 160), stage II (*n* = 32), stage III (*n* = 49), and stage IV (*n* = 3) were determined. The bronchoscopy (49/244, 20%), percutaneous histological biopsy (122/244, 50%), endobronchial ultrasound (EBUS) (24/244, 10%), and surgery of a metastatic site (49/244, 20%) identified the histological subtypes in lung cancer patients (Table 2). All lung cancer patients who participated in this study were treatment-naïve.

Baseline characteristics, including age and percentage of the male gender, are presented in Table 3. The levels four biomarkers including, CIC, CYFRA 21-1, cTnT, and NT-proBNP, were measured with 9G test™ Cancer/Lung test. The levels of CIC, CYFRA 21-1, cTnT, NT-proBNP, CIC/CYFRA 21-1, cTnT/NT-proBNP, and lung cancer index (LC index) with mean values and standard deviation for healthy individuals and cancer patients are summarized in Table 3. 

The CIC levels were slightly higher in lung cancer patients (5.16 (±7.46) ng/mL) than those of a healthy population (4.47 (±5.22) ng/mL). The levels of CYFRA 21-1 were slightly lower in lung cancer patients (2.33 (±2.78) ng/mL) than in a healthy population (3.89 (±4.84) ng/mL). These results indicate that the anti-CYFRA 21-1 autoantibody levels increase in cancer patients. The anti-CYFRA 21-1 autoantibodies complexes with CYFRA 21-1 to form CIC in cancer patients. Hence, the levels of free CYFRA 21-1 in cancer patients are slightly lower than in healthy individuals. The cardiac biomarker cTnT also showed a marked increase in cancer patients (22.4 (±18.8) pg/mL) than the healthy population (16.7 (±13.6) pg/mL). The NT-proBNP levels were slightly lower in cancer patients as compared to healthy individuals.

**Figure 1:**
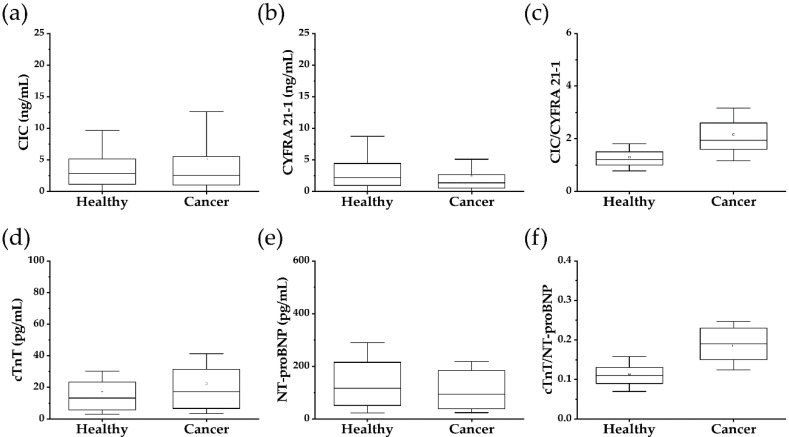
Discrimination of healthy control and lung cancer patients by using (**a**) CIC; (**b**) CYFRA 21-1; (**c**) CIC/CYFRA 21-1 ratio; (**d**) cTnT; (**e**) NT-proBNP; and (**f**) cTnT/NT-proBNP ratio. Healthy control, *n* = 359; lung cancer samples, *n* = 224.

The Mann–Whitney U test allowed determining the differences in biomarker levels between a healthy population and cancer patients (Table 3). The *p*-value < 0.05 indicates a significant difference in the levels of biomarkers in healthy individuals and cancer patients. Mann–Whitney U test demonstrated that the levels of CYFRA 21-1 (*p* < 0.0001) and cTnT (*p* < 0.0015) in cancer patients and healthy population are significantly different. However, these differences were not significant for cancer patients’ discrimination from the healthy population (Figure 1).

Lung cancers can be broadly classified into two major subtypes as small cell lung carcinoma and non-small cell lung carcinoma (NSCLC), which are further divided into the subtypes. It is crucial for a biomarker-based lung cancer test to identify all lung cancer subtypes with high sensitivity and specificity. The subtype-specific levels of the biomarkers used in this study were analyzed (Table S1). The subtype-specific LC index values for mucinous adenocarcinoma (*n* = 4), squamous cell carcinoma (keratinizing) (*n* = 8), mucoepidermoid carcinoma (*n* = 1), small cell carcinoma (*n* = 2) were much lower than the other subtypes of the cancers. Hence, some of the samples from these categories showed false-negative results. It is known that squamous cell carcinomas are typically less problematic to diagnose because they can be detected by bronchoscopy. However, the diagnosis of peripheral lung adenocarcinomas is more challenging. Therefore, identifying lung adenocarcinomas by using biomarkers is crucial for the early treatment of cancer patients. As shown in Table 3 and Table S1, the LC index values are about two folds higher in cancer patients than in the healthy population. Hence, the LC index used in this study detects the types and subtypes of lung cancers with high efficiency.

To determine these biomarkers’ applicability in identifying cancer patients at a specificity of 97.3%, we determined the sensitivities (Table 4). As shown in Figure 1 and Table 4, the levels of CYFRA 21-1, cTnT, and NT-proBNP alone failed to detect cancer patients at the specificity level of 97.3%. The CIC levels could detect cancer patients with a sensitivity of 6.60% and 97.3% specificity, which is not significant for the clinical application.

The fact that the anti-CYFRA 21-1 autoantibody levels are higher in cancer patients than in healthy individuals leads us to apply the CIC/CYFRA 21-1 ratio to discriminate between cancer patients and healthy individuals. It is evident from the data in Table 4 and Figure 1c that the use of CIC/CYFRA 21-1 ratio allowed us to improve the sensitivity to 29.5% at 97.3% specificity. Similarly, as reported earlier, that the levels of cTnT are elevated in cancer patients than in the healthy population lead us to a cTnT/NT-proBNP ratio. As shown in Table 4 and Figure 1f, the cTnT/NT-proBNP ratio discriminated the cancer patients and healthy individuals with a 37.4% sensitivity at 97.3% specificity. Though these sensitivities and specificities are comparable to many commercial assays, the use of these ratios are not significant to be able to use as a companion to LDCT.

**Figure 2:**
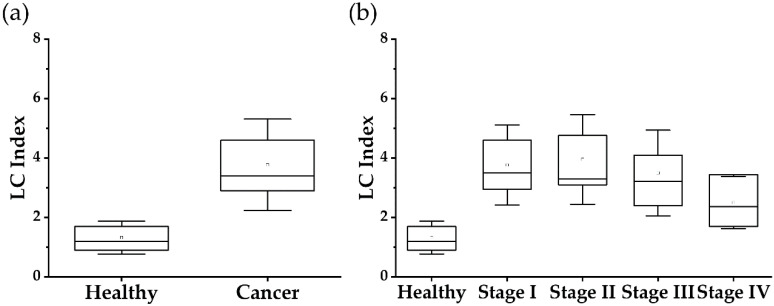
Application of LC index in (**a**) Discrimination of healthy control (*n* = 359) and all stage lung cancer patients (*n* = 244) (**b**) Discrimination of healthy control (*n* = 359) and stage I (*n* = 160), stage II (*n* = 32), stage III (*n* = 49), stage IV (*n* = 3) lung cancer patients.

Therefore, we combined the CIC/CYFRA 21-1 ratio and cTnT/NT-proBNP ratio to generate the LC index according to Equation (1) (see the experimental). As shown in Table 4 and Figure 2a, the LC index allows us to identify cancer patients from the healthy population with a 75.0% sensitivity at 97.3% specificity. According to our knowledge, this is the first ever report that detects stage 0~IV cancers with a 75.0% sensitivity at 97.3% specificity.

It is crucial to notice that the method presented in this article allows the detection of stage I, stage II, stage III, and stage IV cancers with the sensitivities of 77.5%, 78.1%, 67.4%, and 33.3%, respectively, at the specificity of 97.3% (Figure 2b, Table 5).

The LC index’s sensitivity for detecting stage IV cancer was slightly low compared to stage I–III. One of the reasons behind this is that there were only three stage IV cancer samples in this study. We understand that an additional clinical study is warranted to evaluate the presented method for detecting stage IV lung cancer patients.

**Figure 3:**
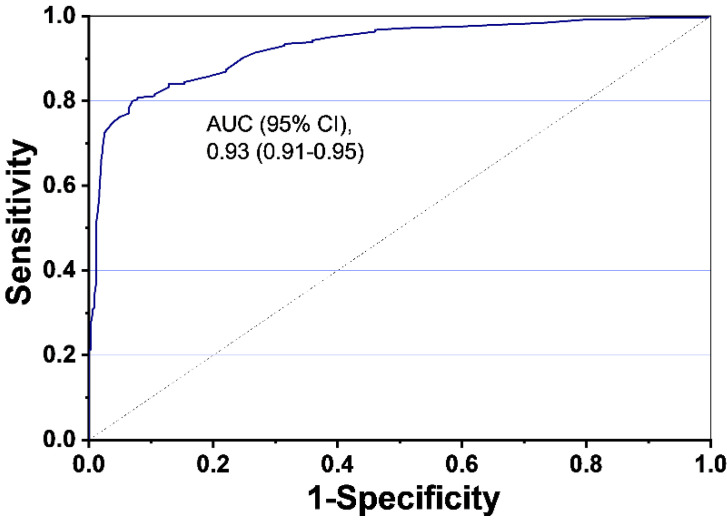
Receiver Operating Characteristic (ROC) curve analysis using the LC index values (*n* = 603).

The population-weighted area under curve (AUC) was found to be 0.93 (95% CI, 0.91–0.95) (Figure 3) for the 9G test^TM^ Cancer/Lung that uses LC index values to discriminate between cancer patients and healthy individuals. 

## 3. Discussion

LDCT is a promising mass screening method for the early diagnosis of lung neoplasms. Several studies have provided substantial evidence that the LDCT is six times superior to CXR in terms of accuracy and sensitivity. The application of LDCT in the mass screening of lung cancer has proved beneficial in identifying stage 0~I lung cancers and improving the 5-year survival rate in lung cancer patients. It was evident from the clinical studies on lung cancer screening that lung cancer screening using LDCT demonstrates a 20% reduction in lung cancer mortality and a 7% reduction in all-cause mortality compared to CXR. With several advantages, LDCT is the best lung cancer screening method to date. However, the only drawback of LDCT is that 96% of LDCT positive patients are confirmed as false-positive upon biopsy. Hence, to reduce the false-positive rate of LDCT and detect stage 0~I lung cancer patients in the general population, a test that can be complementary to the LDCT is highly desirable. Even though the biopsy is an ultimate answer, screening many LDCT positive samples by biopsy is an unreasonable task. Therefore, a blood-based biomarker test based on non-invasive cancer diagnostics principles is a highly desirable companion for LDCT. Such a biomarker test can be used in combination with LDCT for lung cancer screening to improve the diagnostic capacities and reduce false-positive rates of LDCT.

To provide a concrete answer to a decade long problem in cancer screening, we proposed using a 9G test^TM^ Cancer/Lung, a test that allows diagnosis of stage 0~IV lung cancers. 9G test^TM^ Cancer/Lung uses four biomarkers’ simultaneous detection and generates a lung cancer index used to discriminate healthy individuals and cancer patients with 97.3% specificity. According to our recent report on lung cancer detection by quantification of CYFRA 21-1 and CYFRA 21-1-Anti-CYFRA 21-1 autoantibody immune complex for the detection of early-stage lung cancer, we found it is possible to detect stage 0~1 lung cancers with the right set of a biomarker panel.

Several tumor markers have been studied for the detection of symptomatic cancer (stage II-IV) in patients. The c-reactive protein (CRP), carbohydrate antigen 19-9 (CA19-9), carbohydrate antigen 15-3 (CA15-3), carcinoembryonic antigen (CEA), and cancer antigen 125 (CA125) are the majority of tumor markers used for the detection of lung cancers [38,39]. Unfortunately, the low sensitivity (10–60%) and specificity (52–86%) of these biomarkers make them unsuitable for the diagnosis of stage 0~I lung cancer [40,41]. The C-terminus of cytokeratin 19 (CYFRA 21-1) is a lung-specific marker with the sensitivity and specificity of 43% and 89%, respectively, that are very low for lung cancer screening [42]. The broad and overlapping range of tumor marker levels in a healthy population and lung cancer cases is one reason behind these biomarkers’ low sensitivity and specificity.

On the contrary, the tumor marker-specific autoantibodies show 5–10 times higher levels in cancer patients than in a healthy population [43]. Interestingly, in our previous study, we found that the plasma levels of CYFRA 21-1-Anti-CYFRA 21-1 autoantibody immune complex (CIC) were at higher levels in lung cancer patients than in the healthy population. However, CIC’s plasma levels were at lower levels compared to the free CYFRA 21-1 in a healthy population than in lung cancer patients [37]. Therefore, it was assumed that using these two biomarkers in lung cancer detection could allow high sensitivity and specificity. However, we also recognized that to achieve higher sensitivity and specificity requires the use of multiple biomarkers [44,45]. The literature searches on cardiac biomarkers exhibited that cardiac troponin T (cTnT) levels are higher in treatment-naïve cancer patients than healthy individuals [30,31,32]. cTnT levels in cancer patients are elevated due to the cancer-associated arterial micro-thrombosis promoted by neutrophil extracellular traposis, and due to the elevated levels of cancer-specific autoantibodies [33,34]. The levels of N-terminal pro-brain natriuretic peptide (NT-proBNP), another cardiac biomarker, show substantial overlaps in the healthy population and cancer patients [35]. Hence, for the development of the 9G test^TM^ Cancer/Lung test, we chose a panel of four biomarkers, including CIC, CYFRA 21-1, cTnT, and NT-proBNP. These biomarkers were used to determine lung cancer index that effectively discriminated between the healthy individuals and stage 0~IV lung cancer patients.

As indicated by the presented data, the LC index determined by 9G test^TM^ Cancer/Lung effectively detects stage 0~IV lung cancers with a sensitivity and specificity of 75.0% (69.1~80.3) and 97.3% (95.0~98.8), respectively. Furthermore, the 22 samples that were identified as cancer in LDCT were correctly identified 22 samples as negative by the 9G test^TM^ Cancer/Lung test, indicating a 100% concordance with biopsy. The detection of stage I, stage II, stage III, and stage IV cancers with the sensitivities of 77.5%, 78.1%, 67.4%, and 33.3%, respectively, at the specificity of 97.3%, have never been reported before. According to our knowledge, this is the first ever report that detects stage 0~IV cancers with a 75.0% sensitivity at 97.3% specificity. Therefore, obtained results indicate that the use of 9G test^TM^ Cancer/Lung with LDCT for lung cancer screening can increase stage I cancer detection, which is crucial to improve the currently low 5-year survival rates [46,47]. Hence, these results indicate that the 9G test^TM^ Cancer/Lung test has a high potential to reduce the false-positive rate of LDCT if used in conjunction with LDCT. 

It is crucial to note that the cardiovascular risk factors and smoking as a risk factor of lung cancer were not considered in this study. It is reported earlier that the integrated risk prediction model that combined smoking exposure increases the sensitivity of the test [48]. Hence, further study is required to add cardiac risk factors and smoking exposure as additional parameters for improving the presented method’s sensitivity.

## 4. Materials and Methods

### 4.1. Study Population

Clinical samples from healthy individuals (*n* = 359) and lung cancer patients (*n* = 244) were collected and tested at respective locations in South Korea (Table 1). All healthy individuals and cancer patients were screened by LDCT, followed by the biopsy for individuals whose results were LDCT positive. An amount of 175/359 clinical samples from healthy individuals were provided by the Nowon Eulji Medical Center, Eulji University, Seoul, South Korea. Ethical Clearance Committee on Human Rights Related to Research Involving Human Subjects of Nowon Eulji Medical Center, Eulji University, Seoul, South Korea, approved this study (2020-EC-01-008). In total, 8/175 samples obtained at this location were found to be LDCT positive and were confirmed as negative in the biopsy. About 120/359 samples from healthy individuals and 50/244 samples from cancer patients were obtained from the Korea Cancer Central Hospital, Korea Institute of Radiological & Medical Sciences, Seoul, Korea. The Ethical Clearance Committee on Human Rights Related to Research Involving Human Subjects of Korea Cancer Central Hospital, Korea Institute of Radiological & Medical Sciences, Nowon-gu, Seoul, South Korea, approved this study (KIRAMS 2018-10-006). The Ajou Human Bio-Resource Bank (AHBB), a member of the National Biobank of Korea, supported by the Ministry of Health and Welfare, provided 64/359 specimens from healthy individuals for this study. All samples derived from the National Biobank of Korea were obtained with informed consent under institutional review board-approved protocols (AJHB-2019-28). Overall, 14/64 of samples at this site were identified as LDCT positive but were confirmed to be negative in the biopsy. The Biobank of Gyeongsang National University Hospital (a member of Korea Biobank Network), Jinju-si, South Korea provided 12/244 bio-specimens from cancer patients and related data used in this study. All samples derived from the Biobank of Gyeongsang National University Hospital were obtained with informed consent under institutional review board-approved protocols (2019-021). The 182/244 bio-specimen and data used in this study were provided by Asan Bio-Resource Center, Korea Biobank Network, Seoul, South Korea. All samples derived from the Asan Bio-Resource Center were obtained with informed consent under institutional review board-approved protocols (2019-14(193)). Venous blood samples were collected, and local laboratory standard procedures were followed for on-site analysis. We recorded the levels of CIC, CYFRA 21-1, cTnT, and NT-proBNP in the plasma samples of healthy individuals and cancer patients.

### 4.2. Determination of Lung Cancer Index Using 9G Test™ Cancer/Lung Test

The measurements of CIC, CYFRA 21-1, cTnT, and NT-proBNP in the plasma samples of healthy individuals and cancer patients were performed by the 9G test™ Cancer/Lung test (Biometrix Technology Inc., Chuncheon, South Korea). The 9G test™ Cancer/Lung test quantifies plasma levels of CIC, CYFRA 21-1, cTnT, and NT-proBNP at room temperature in 30 min in the analytical detection range of 0.05–5 ng/mL (LoD = 0.05 ng/mL), 0.05–5 ng/mL (LoD = 0.04 ng/mL), 1–120 pg/mL (LoD = 0.87 pg/mL), and 7.0–600.0 pg/mL (LoD = 3.7 pg/mL), respectively. The CV for the detection of these biomarkers was less than 10% in the whole detection range. The biomarker levels were analyzed for the cut-off levels with 97.3% specificity in the discrimination of healthy population and cancer patients. The values of the CIC/CYFRA 21-1 ratio and cTnT/NT-proBNP ratio were also used determined for the discrimination of healthy and cancer patients. Finally, the lung cancer index (LC index) was determined according to Equation (1) and used to discriminate between a healthy population and cancer patients with 97.3% specificity.

LC index = [(CIC/CYFRA 21-1) × (cTnT/NT-proBNP)](1)

### 4.3. Statistical Analysis

Continuous data are expressed as the mean ± standard deviation (SD) or median with interquartile range (IQR) as required. Categorical data are presented as counts and percentages. The difference between healthy individuals and cancer patients of different cancer types was calculated using the Mann–Whitney U test. For all analyses, two-sided *p* values < 0.05 were considered to indicate statistical significance. The sensitivity, specificity, positive predictive value (PPV), and negative predictive value (NPV) at a 95% confidence interval (CI) were calculated. Statistical analyses were performed using Medcalc for Windows version 17.4.4 (Medcalc Software, Mariakerke, Belgium).

## 5. Conclusions

To the best of our knowledge, this is the first report on a method that, if used in combination with LDCT for lung cancer screening, can improve the diagnostic capacities and reduce false-positive rates of LDCT. Furthermore, it is a unique report on the use of a panel of four biomarkers for the detection of stage 0~IV lung cancer patients with very high sensitivity and specificity than any other reported methods. The presented method detects lung cancers, with 75.0% (69.1~80.3) sensitivity and 97.3% (95.0~98.8) specificity. The sensitivities for the detection of stage I, II, III, and IV lung cancers were 77.5%, 78.1%, 67.4%, and 33.3%, respectively, at the specificity of 97.3%. According to our knowledge, this is the first-ever report that detects stage 0~IV cancers with a 75.0% sensitivity at 97.3% specificity. The cardiovascular risk factors, smoking as a risk factor of lung cancer, were not integrated into the data analysis. Consequently, further study is essential to add cardiac risk factors and smoking exposure as additional parameters to evaluate the presented method. Nevertheless, the ROC analysis showed the population-weighted AUC of 0.93 (95% CI, 0.91–0.95). Therefore, the method presented here can be applied to identify lung cancer patients (stage 0~IV) from a seemingly healthy population. The obtained results indicate that the use of the presented test with LDCT for lung cancer screening can (i) reduce the false-positive rate of LDCT, and (ii) increase the rate of stage I cancer detection, which is crucial to improve the currently meager 5-year survival rate in lung cancer patients.

## Figures and Tables

**Table 1:** Clinical samples (*n* = 603) obtained from healthy individuals (*n* = 359) and cancer patients (*n* = 244).

Sources of Clinical Samples	Healthy Individuals	Lung Cancer Patients *	IRB No.
Nowon Eulji Medical Center, Eulji University, Seoul, South Korea	175 ^a^	-	2020-EC-01-008
Korea Cancer Central Hospital, Seoul, South Korea	120	50	KIRAMS 2018-10-006
Ajou Human Bio-Resource Bank (AHBB)	64 ^b^	-	AJHB-2019-28
Biobank of Gyeongsang National University Hospital,	-	12	2019-021
Asan Bio-Resource Center, Korea Biobank Network	-	182	2019-14(193)
Total	359	244	-

^a^ 8/175 samples were LDCT positive but found to be negative in biopsy; ^b^ 14/64 samples were LDCT positive but found to be negative in biopsy; * all samples were LDCT positive and confirmed to be lung cancer in biopsy; IRB, the institutional review board.

**Table 2:** Types, subtypes, and stage-wise distribution of lung cancer (*n* = 244) samples.

Lung Cancer Type	Subtypes	Lung Cancer Stages			
		Stage I	Stage II	Stage III	Stage IV
Adenocarcinoma	Adenoid cystic carcinoma	1		2	
	Bronchioloalveolar adenocarcinoma	36	3	5	
	Mucinous adenocarcinoma	4			
	Adenocarcinoma	43	16	9	2
	Papillary adenocarcinoma	40	2	8	
Squamous cell carcinoma	Adenosquamous carcinoma	2	2	3	
	Squamous cell carcinoma	18	3	8	
	Squamous cell carcinoma, keratinizing	4		4	
	Squamous cell carcinoma, large cell, non-keratinizing	4	2	1	
	Mucoepidermoid carcinoma	1			
Small Cell	Combined small cell carcinoma	1	1	1	1
	Small cell carcinoma	2			
Large cell carcinoma	Large cell carcinoma	2	2	4	
	Large cell carcinoma with neuroendocrine feature			2	
Non-small cell carcinoma	Bronchogenic non-small cell carcinoma	1	1	1	
	Sarcomatoid carcinoma	1		1	

**Table 3:** Characteristics of the Study Participants (*n* = 603).

Characteristic	Healthy Population (*n* = 359)	Cancer Patients (*n* = 244)	*p*-Value (Healthy vs. Cancer)
Age, years (SD)	57.3 (±12.0)	62.7 (±9.30)	-
Male gender, *n* (%)	140 (38.9)	161 (65.9)	-
CIC (pg/mL; SD)	4.47 (±5.22)	5.16 (±7.46)	0.7056
CYFRA 21-1 (pg/mL; SD)	3.89 (±4.84)	2.33 (±2.78)	0.0001
cTnT (pg/mL; SD)	16.7 (±13.6)	22.4 (±18.8)	0.0015
NT-proBNP (pg/mL; SD)	155.6 (±133.6)	121.7 (±97.5)	0.005
CIC/CYFRA 21-1 (SD)	1.290 (±0.52)	2.16 (±1.00)	0.0001
cTnT/NT-proBNP (SD)	0.11 (±0.04)	0.19 (±0.06)	0.0001
LC Index (SD)	1.45 (±0.73)	3.90 (±1.87)	0.0001

CIC, CYFRA 21-1-Anti-CYFRA 21-1 autoantibody immune complex; CYFRA 21-1-Anti-CYFRA 21-1 autoantibody immune complex; cTnT, cardiac troponin T; NT-proBNP, N-terminal pro-brain natriuretic peptide; LC Index, lung cancer index; Variables are displayed as mean and standard deviation (SD); Difference between healthy and patients with different cancer types was calculated using the Mann–Whitney U test. *p* values < 0.05 were considered to indicate statistical significance.

**Table 4:** Clinical efficiency in terms of sensitivity, specificity, PPV, and NPV of LC index for discrimination of healthy individuals (*n* = 359) and cancer patients (*n* = 244).

Variable	Sensitivity (95% CI)	Specificity (95% CI)	PPV (95% CI)	NPV (95% CI)
CIC	6.60 (3.80~10.4)	97.3 (95.0~98.8)	64.0 (44.4~79.8)	59.0 (58.0~59.9)
CYFRA 21-1	0.00 (0.00~1.50)	97.3 (95.0 ~98.8)	0.00	57.3 (57.0~57.8)
cTnT	0.00 (0.00~1.50)	97.3 (95.0~98.8)	0.00	57.3 (57.0~57.8)
NT-proBNP	0.00 (0.00~1.50)	97.3 (95.0~98.8)	0.00	57.3 (57.0~57.8)
CIC/CYFRA 21-1	29.5 (23.9~35.7)	97.3 (95.0~98.8)	89.0 (80.3~94.0)	65.6 (63.7~67.4)
cTnT/NT-proBNP	34.0 (28.0~40.3)	97.3 (95.0~98.8)	90.2 (82.6~94.8)	67.0 (65.0~69.0)
LC Index	75.0 (69.1~80.3)	97.3 (95.0~98.8)	95.4(91.4~97.5)	84.3 (81.2~87.0)

PPV, positive predictive value; NPV, negative predictive value; CI, confidence interval.

**Table 5:** The efficiency of the LC index for the detection of stage I~stage IV lung cancer patients.

Cancer Stages (*n*)	Sensitivity (95% CI)	Specificity (95% CI)	PPV (95% CI)	NPV (95% CI)
Stage I (*n* = 160)	77.5 (70.2~83.8)	97.3 (95.0~98.8)	93.2 (87.8~96.4)	90.1 (87.2~92.4)
Stage II (*n* = 32)	78.1 (60.0~90.0)	97.3 (95.0~98.8)	73.5 (58.7~84.5)	97.9 (96.0~99.0)
Stage III (*n* = 49)	67.4 (52.5~80.0)	97.3 (95.0~98.8)	78.6 (65.2~87.8)	95.4 (93.2~96.8)
Stage IV (*n* = 3) *	33.3 (0.80~90.6)	97.3 (95.0~98.8)	10.0 (2.00~38.4)	99.4 (98.7~99.7)

* the sensitivity for stage IV cancer determination would require more number of samples to improve accuracy.

## Supplementary Materials

The following are available online at https://www.mdpi.com/2072-6694/12/11/3192/s1, Table S1: Types and subtypes specific distribution of levels of biomarkers in lung cancer samples (*n* = 244).
