# Supplementary material for: 9G TestTM Cancer/Lung: A Desirable Companion to LDCT for Lung Cancer Screening
Source: Cancers (Basel). 2020 Oct 30;12(11):3192. doi: 10.3390/cancers12113192 (PMC7692999; doi:10.3390/cancers12113192)
Supplement: Supplementary file 1 [file cancers-12-03192-s001.pdf]

Supplementary Materials

9G Test™ Cancer/Lung: A Desirable Companion to LDCT for Lung Cancer Screening

Wonho Choe, Jeong Don Chae, Byoung-Hoon Lee, Sang-Hoon Kim, So Young Park, Satish Balasaheb Nimse, Junghoon Kim, Shrikant Dashrath Warkad, Keum-Soo Song, Ae-Chin Oh, Young Jun Hong and Taisun Kim

Table S1. Types and subtypes specific distribution of levels of biomarkers in lung cancer samples (*n* = 244).

Lung Cancer type	Subtypes	Number of Samples	Biomarker levels						
			CIC (pg/mL; SD)	CYFRA 21-1 (pg/mL; SD)	CIC/ CYFRA 21-1 (SD)	cTnT (pg/mL; SD)	NT-proBNP (pg/mL; SD)	cTnT/ NT-proBNP (SD)	LC Index (SD)
**Adenocarcinoma**	Adenoid cystic carcinoma	3	12.6 (16.4)	4.02 (4.82)	2.60 (0.81)	30.5 (18.7)	172.4 (84.0)	0.17 (0.04)	4.48 (2.37)
	Bronchioloalveolar adenocarcinoma	44	2.41 (2.10)	1.29 (1.08)	2.19 (1.59)	19.07 (17.1)	115.6 (98.8)	0.17 (0.07)	3.45 (1.84)
	Mucinous adenocarcinoma	4	69.0 (10.2)	3.89 (3.66)	1.48 (1.12)	2.73 (1.68)	32.0 (19.82)	143.0 (104.3)	0.25 (0.09)
	Adenocarcinoma	70	4.67 (5.94)	2.16 (2.41)	2.20 (0.81)	26.2 (21.5)	138.4 (103.0)	0.18 (0.06)	3.91 (1.71)
	Papillary adenocarcinoma	50	2.50 (1.79)	1.30 (0.98)	2.05 (0.78)	22.3 (19.5)	115.1 (99.2)	0.20 (0.06)	4.11 (2.07)
**Squamous cell carcinoma**	Adenosquamous carcinoma	7	4.18 (8.50)	1.57 (2.59)	1.91 (0.74)	13.4 (12.2)	67.7 (43.5)	0.18 (0.07)	3.63 (2.48)
	Squamous cell carcinoma	29	14.6 (12.4)	6.30 (4.13)	2.07 (0.81)	23.1 (18.45)	126.8 (108.6)	0.20 (0.04)	4.00 (1.48)
	Squamous cell carcinoma, keratinizing	8	2.11 (1.32)	1.21 (0.51)	1.74 (0.74)	13.5 (9.64)	81.4 (38.8)	0.16 (0.09)	2.69 (1.57)
	Squamous cell carcinoma, large cell, non-keratinizing	7	2.71 (3.00)	0.98 (1.05)	2.53 (0.51)	17.0 (15.9)	104.4 (104.5)	0.18 (0.05)	4.56 (1.67)
	Mucoepidermoid carcinoma	1	8.4 (-)	5.0 (-)	1.7 (-)	10.2 (-)	65.6 (-)	0.15 (-)	2.6 (-)
**Small Cell**	Combined small cell carcinoma	4	3.00	1.29	2.40	17.2	114.1	0.15	3.68

			(1.08)	(0.45)	(0.97)	(6.72)	(30.5)	(0.04)	(1.87)
	Small cell carcinoma	2	1.37<br>(1.07)	0.99<br>(0.74)	1.35<br>(0.07)	11.9<br>(9.04)	56.2<br>(25.8)	0.20<br>(0.08)	2.70<br>(1.13)
**Large cell carcinoma**	Large cell carcinoma	8	4.72<br>(6.35)	2.04<br>(2.47)	2.33<br>(0.61)	21.0<br>(21.8)	121.5<br>(107.4)	0.16<br>(0.05)	3.55<br>(1.05)
	Large cell carcinoma with neuroendocrine feature	2	7.34<br>(2.49)	4.30<br>(1.81)	1.75<br>(0.15)	22.2<br>(15.0)	131.1<br>(102.1)	0.18<br>(0.02)	3.14<br>(0.71)
**Non-small cell carcinoma**	Bronchogenic non-small cell carcinoma	3	17.1<br>(10.4)	6.05<br>(1.10)	2.73<br>(1.30)	26.6<br>(16.9)	128.3<br>(75.5)	0.20<br>(0.03)	5.66<br>(2.98)
	Sarcomatoid carcinoma	2	16.5<br>(22.6)	5.83<br>(8.07)	3.51<br>(0.98)	32.45<br>(37.6)	140.1<br>(156.9)	0.22<br>(0.02)	7.51<br>(1.26)

© 2020 by the authors. Licensee MDPI, Basel, Switzerland. This article is an open access article distributed under the terms and conditions of the Creative Commons Attribution (CC BY) license (http://creativecommons.org/licenses/by/4.0/).
